# TIR-Domain-Containing Adaptor-Inducing Interferon-*β* (TRIF) Is Involved in Glucose Metabolism in Adipose Tissue through the Insulin/AKT Signaling Pathway

**DOI:** 10.1155/2020/6942307

**Published:** 2020-12-09

**Authors:** Junling Yang, Ken-Ichiro Fukuchi

**Affiliations:** Department of Cancer Biology and Pharmacology, University of Illinois College of Medicine Peoria, 1 Illini Drive, Peoria, IL 61605, USA

## Abstract

Obesity significantly increases the risk of developing type 2 diabetes mellitus and other metabolic diseases. Obesity is associated with chronic low-grade inflammation in white adipose tissues, which is thought to play an essential role in developing insulin resistance. Many lines of evidence indicate that toll-like receptors (TLRs) and their downstream signaling pathways are involved in development of chronic low-grade inflammation and insulin resistance, which are associated with obesity. Mice lacking molecules positively involved in the TLR signaling pathways are generally protected from high-fat diet-induced inflammation and insulin resistance. In this study, we have determined the effects of genetic deficiency of toll/interleukin-1 receptor-domain-containing adaptor-inducing interferon-*β* (TRIF) on food intake, bodyweight, glucose metabolism, adipose tissue macrophage polarization, and insulin signaling in normal chow diet-fed mice to investigate the role of the TRIF-dependent TLR signaling in adipose tissue metabolism and inflammation. TRIF deficiency (TRIF^−/−^) increased food intake and bodyweight. The significant increase in bodyweight in TRIF^−/−^ mice was discernible as early as 24 weeks of age and sustained thereafter. TRIF^−/−^ mice showed impaired glucose tolerance in glucose tolerance tests, but their insulin tolerance tests were similar to those in TRIF^+/+^ mice. Although no difference was found in the epididymal adipose mass between the two groups, the percentage of CD206^+^ M2 macrophages in epididymal adipose tissue decreased in TRIF^−/−^ mice compared with those in TRIF^+/+^ mice. Furthermore, activation of epididymal adipose AKT in response to insulin stimulation was remarkably diminished in TRIF^−/−^ mice compared with TRIF^+/+^ mice. Our results indicate that the TRIF-dependent TLR signaling contributes to maintaining insulin/AKT signaling and M2 macrophages in epididymal adipose tissue under a normal chow diet and provide new evidence that TLR4-targeted therapies for type 2 diabetes require caution.

## 1. Introduction

Insulin resistance significantly raises the risk of developing metabolic diseases such as type 2 diabetes mellitus (T2DM), hypertension, coronary heart disease, Alzheimer's disease (AD), and cerebrovascular disease (CVD), as well as certain infectious disease, such as COVID-19 [1–5]. Excess calories caused by lasting imbalance between energy intake and expenditure are predominantly stored in visceral (gonadal in rodents) and subcutaneous white adipose tissue (WAT) [6]. Visceral adipose tissue of obese subjects shows low-grade chronic inflammation that is characterized by increases in proinflammatory immune cells such as M1 macrophages and Th1 cells and/or by decreases in anti-inflammatory immune cells such as M2 macrophages and Treg cells. These inflammatory changes in visceral adipose tissue have been identified as a major contributor to insulin resistance in obese subjects [7, 8]. Accordingly, identification of the signaling mechanisms and effectors that regulate adipose tissue macrophage polarization is essential to establishing therapeutic measures for obesity and insulin resistance.

Toll-like receptors (TLRs) are a class of pattern recognition receptors (PRRs) that sense pathogen-associated molecular patterns, structurally conserved molecules of microorganisms, damage-associated molecular patterns, and molecules released from injured host cells. Activation of TLRs through PRRs induces innate immune responses as well as, ultimately, adaptive immune responses, which are thought to be causally linked to metabolic diseases [9]. To date, at least 13 different TLRs have been reported [10]. TLR1, TLR2, TLR4, TLR5, TLR6, and TLR10 are found on cellular membranes. TLR3, TLR7, TLR8, and TLR9 are localized to endosomal compartments [11]. With an exception of TLR3, all TLRs signal through an adaptor, myeloid differentiation factor 88 (MyD88), to activate NF-*κ*B and mitogen-activated protein (MAP) kinases (MyD88-dependent pathway), while TLR3 and TLR4 can mediate their signals via an adaptor, toll-interleukin-1 receptor (TIR)-domain-containing adaptor-inducing interferon-*β* (TRIF), to activate interferon regulatory factor 3 (IRF3) (MyD88-independent/TRIF-dependent pathway) [12]. Increasing lines of evidence indicate that TLR signaling plays an important role in development of insulin resistance. Several genetic studies, for instance, found that certain alleles of TLR genes are linked to specific markers of glucose metabolism [13–16]. Female mice with skeletal muscle-specific MyD88 deficiency are protected from inactivity-induced adiposity and insulin resistance [17, 18]. Mice with hepatocyte-specific MyD88 deficiency are prone to develop glucose intolerance, inflammation, and hepatic insulin resistance regardless of bodyweight [19]. TRIF deficiency can prevent development of type 1 diabetes in nonobese diabetic mice by changing the gut microbiota composition and suppression of dendritic cells [20]. Gestational diabetes mellitus in placentae and maternal hyperglycemia are positively associated with increased TLR4/MyD88/NF-*κ*B signaling [21]. Insulin resistance in skeletal muscles from nonobese patients with impaired glucose tolerance is mainly mediated by upregulation of TLR4 due to increased IL-6-mediated STAT3 activation [22]. Mice with TRIF deficiency show glucose intolerance that is associated with pancreatic *β*-cell dysfunction in vitro [23].

Adipose tissue TLR signaling in association with obesity and glucose metabolism has been reported in several studies. Expression of TLR9 is upregulated during adipocyte differentiation and expressed in murine gonadal and human visceral adipocytes. Activation of TLR9 by its ligands inhibits proinflammatory resistin secretion and TLR9 deficiency or knockdown decreases expression of anti-inflammatory adiponectin expression in mice or cultured adipocytes, respectively, suggesting that adipocytic TLR9 signaling protect inflammation associated with obesity [24]. Metabolic stress induces activation of TLR4 signaling in immune cells, leading to release of proinflammatory cytokines for recruitment of macrophages to adipose tissue, potentially exacerbating chronic inflammation and insulin resistance [25]. TLR4-mediated activation of both MyD88 and TRIF signaling pathways is required for production of CD11c^+^ M1 macrophages in adipose tissues during a high-fat diet (HFD)-induced macrophage polarization [26]. Statins reduce increased expression of inflammatory cytokines such as IL-6 and CCL2 in adipose tissues in obese (ob/ob) mice, which is thought to be mediated by suppression of the TLR4/TRIF/IRF3/IFN*β* signaling pathway by statins but not by the TLR4/MyD88/NF-kB pathway [27]. MyD88- or TRIF-deficient mice are protected against lard (saturated lipids) diet-induced adipose tissue inflammation and insulin resistance [28]. However, the role of TRIF in glucose metabolism in adipose tissue has not been investigated under normal chow diet. In this study, we determined the effects of TRIF deficiency on food intake, bodyweight, glucose metabolism, adipose tissue macrophage polarization, and adipose tissue insulin signaling in normal chow diet-fed mice to investigate the role of the TRIF-dependent pathway in adipose tissue metabolism and inflammation.

## 2. Materials and Methods

### 2.1. Experimental Animals

TRIF-deficient (TRIF^−/−^) mice [29] were obtained from Dr. Alan Aderem, Institute for Systems Biology and backcrossed to C57BL/6J mice more than 10 generations. The genotyping for the TRIF gene was performed by PCR of DNA extracted from mouse tails using the Extract-N-Amp tissue PCR kit (Sigma-Aldrich, St. Louis, MO). The PCR was carried out in 25 *μ*l volume using 1 *μ*l tail DNA extract and 200 nM each of two primers for the TRIF^−/−^ allele through 35 cycles consisting of 30 s at 94°C, 30 s at 62°C, and 1.5 min at 72°C. The last elongation step was done at 72°C for 10 min. The two primer sequences, forward primer: 5'-GGG TTA GCT GAC TGA CCT GTC CA-3' and reverse primer (neo gene): 5'-CTG CCC ATT CGA CCA CCA AG-3', were used to amplify the TRIF^−^ allele. An additional reverse primer, 5'-CAC TTG TGT CTG GAG CAG CCA-3', was used to amplify the wild-type allele. The PCR products were subjected to electrophoresis through a 1% agarose gel. Mouse tail DNAs extracted from a TRIF^−/−^ and C57BL/6J mouse were used as positive controls for the TRIF^−^ and TRIF^+^ allele, respectively. First, we produced TRIF^+/−^ mice by mating TRIF^−/−^ male mice with C57BL/6J female mice. Next, we mated TRIF^+/−^ male mice with TRIF^+/−^ female mice. The progeny of the latter mating consisted of three different genotypes, TRIF^+/+^, TRIF^+/−^, and TRIF^−/−^. In this study, two groups of male mice with TRIF^+/+^ or TRIF^−/−^ genotypes were used. The mice were housed in groups in cages with bedding and maintained on a 12/12 hr light-dark cycle (on at 06.00 am) under specific pathogen-free condition with ad libitum access to autoclaved food (NIH-31 formula) and water. All animal protocols used for this study were prospectively reviewed and approved by the Institutional Animal Care and Use Committee of the University of Illinois College of Medicine Peoria.

### 2.2. Intraperitoneal Glucose Tolerance Test (IPGTT) and Insulin Tolerance Test (ITT)

At the age of 11.5 months, the mice were conducted for the IPGTT and ITT, as previously described [30, 31]. The animals (*n* = 12/group) were fasted for 16 h, weighed, and intraperitoneally injected with D-glucose (1.5 g/kg) for the IPGTT. Blood was obtained using the tail nick method before and at 30, 60, 90, and 120 min after the D-glucose injection for monitoring glucose levels using a glucometer (ReliOn® Prime, Arkray USA, Minneapolis, MN). Plasma insulin levels were determined by using the insulin ELISA assay kit (Crystal Chem Inc., Downers Grove, IL). The ITT was conducted one week after the IPGTT. Mice were fasted for 4 h, weighed, and intraperitoneally injected with human insulin (0.75 units/kg, Novolin® R, Novo Nordisk, Princeton, NJ). Blood sugar was monitored before and at 15, 30, 60, and 90 min.

### 2.3. Quantification of Plasma Leptin by ELISA

Approximately 100 *μ*l of blood/mouse was drawn from the tail vein at termination by using microhematocrit heparin tubes (Fisher Scientific, Pittsburgh, PA) and centrifuged for 20 min at 2,000 × *g* for plasma isolation. Levels of leptin in plasma were quantified by leptin ELISA kits (Sigma, St. Louis, MO) according to the manufacturer's protocol.

### 2.4. Fluorescence-Activated Cell Sorting (FACS)

Epididymal adipocytes were prepared as previously described [31, 32]. In brief, adipose tissue (*n* = 4/group) was digested at 37°C in PBS (without calcium and magnesium) containing 2% bovine serum albumin (BSA) and 2 mg/ml collagenase VI (Sigma, St. Louis, MO) for 45 min. After undigested fragments were removed using 100 *μ*m filters (Fisher Scientific, Pittsburgh, PA), adipocytes were separated from pellets of stromal vascular fraction cells by centrifugation at 600*g* for 10 min. F4/80-PE (eBioscience, Inc., San Diego, CA), CD11c PerCP-Cyanine5.5 antibodies (eBioscience, Inc., San Diego, CA), and CD206-FITC antibodies (BioLegend, San Diego, CA) were used to identify macrophages, a M1 marker, and a M2 marker, respectively. Labeled cells were loaded and analyzed by FACS (BD Biosciences, San Jose, CA).

### 2.5. Western Blot of Insulin Signaling in White Adipose Tissue, Muscle, and Liver

Insulin signaling in white adipose tissue, muscle, and liver was determined by western blot as previously described [33]. In brief, one week after the IPGTT and ITT, the mice (*n* = 4/group) were fasted overnight. Saline or 5 units/kg of insulin (Novolin® R, Novo Nordisk, Princeton, NJ) was injected intraperitoneally. Ten minutes later, epididymal fat pads, muscle, and livers were quickly removed and snap-frozen in liquid nitrogen and then stored at −80°C in a freezer. For analysis of insulin-stimulated AKT phosphorylation, epididymal fat pads and liver tissues were homogenized in RIPA lysis buffer (Sigma, St. Louis, MO) and muscle in 2× Laemmli buffer containing complete miniprotease inhibitor and phosphatase inhibitor cocktail tablets (Roche Diagnostics Corporation, Indianapolis, IN) and centrifuged at 12,000 × *g* for 10 min at 4°C for collecting supernatants. After determining protein concentrations, the proteins were electrophoresed under reducing conditions in 10% SDS-PAGE gels and transferred to PVDF membranes. The membranes were incubated overnight at 4°C with anti-phospho-AKT and anti-AKT antibody (1 : 1000 dilution) (Cell Signaling Technology, Danvers, MA), and specific bands were visualized by an enhanced chemiluminescence system (Amersham, Arlington Heights, IL). The optical densities of the protein bands were determined by densitometric scanning using an HP Scanjet G3010 Photo Scanner and ImageJ V1.40 (NIH, MD). The optical density of the pAKT band was divided by that of the total-AKT band on the same lane from the same membrane for normalization.

## 3. Results and Discussion

**Figure 1:**
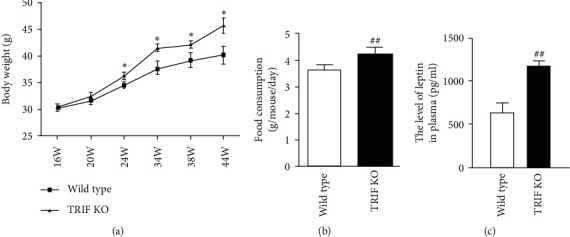
Effects of TRIF deficiency on bodyweight, food consumption, and plasma leptin levels. (a) Animals were weighed weekly from 16 weeks of age through 44 weeks. (b) Food consumption was recorded weekly from 36 weeks through 40 weeks. (c) Plasma samples were harvested at the age of 48 weeks, and plasma leptin was determined by leptin ELISA kit ( ^*∗*^*P* < 0.05,  ^##^*P* < 0.001) (*n* = 12/group).

**Figure 2:**
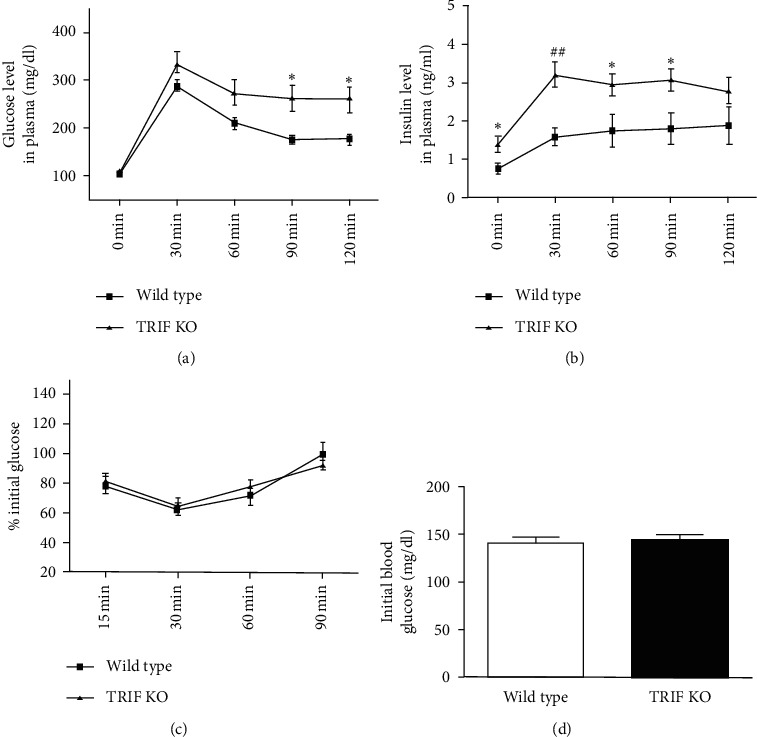
Effects of TRIF deficiency on the IPGTT and ITT. (a) At the age of 46 weeks, mice were fasted for 16 h and injected i.p. with 1.5 g glucose/kg bodyweight, and (b) blood glucose and insulin were measured before and after glucose injection at the indicated time points. (c) At the age of 47 weeks, mice were fasted for 4 h and injected i.p. with 0.75 U insulin/kg bodyweight for the ITT. Blood glucose levels were determined and are shown as percent changes from (d) basal blood glucose levels ( ^*∗*^*P* < 0.05,  ^##^*P* < 0.001) (*n* = 12/group).

Many lines of evidence support the notion that TLR signaling pathways are involved in the development of obesity-associated insulin resistance and adipose tissue inflammation, indicating important roles of innate immunity in metabolic diseases [34]. Patients with obesity and diabetes and obese (ob/ob) mice show increased expression of TLR4 that exhibits an inverse correlation with insulin sensitivity [35]. Development of HFD-induced insulin resistance is prevented in mice with TLR4 deficiency. Although female mice lacking TLR4 are heavier than control TLR4 wild-type female mice, TLR4 deficiency ameliorates insulin resistance in female mice [36]. Interestingly, HFD-induced (58% fat) obesity can be reversed by low-fat diet (10.5% fat) in TLR4 wild-type C57BL/6 mice but not in TLR4-deficient mice [37]. TRIF or MyD88 deficiency prevents lard-induced white adipose tissue inflammation and insulin resistance in mice [28]. However, the effects of TRIF deficiency on bodyweight in detail, food consumption, adipose tissue macrophage polarization, and insulin signaling in normal chow diet-fed mice have not been investigated. We found that TRIF^−/−^ mice were heavier than their wild-type controls. The first significant difference in bodyweight between TRIF^−/−^ and TRIF^+/+^ mice was discernible at age 24 weeks and, thereafter, the difference was maintained during the experimental period (up to 44 weeks of age) (Figure 1(a), *P* < 0.05). Food consumption was measured weekly for one month starting at 9 months of age. TRIF^−/−^ mice consumed more food (4.24 g ± 0.24 g/day/mouse) than TRIF^+/+^ mice (3.64 ± 0.20 g/day/mouse) (Figure 1(b), *P* < 0.01). Therefore, TRIF^−/−^ mice might gain more weight due to increases in food intakes, compared with their wild-type controls. Next, we studied the metabolic phenotype of normal chow diet-fed TRIF^−/−^ mice by carrying out GTTs and ITTs. In the IPGTT, blood glucose levels in TRIF^−/−^ mice were significantly higher than those in TRIF^+/+^ mice at 90 min and 120 min (*P* < 0.05) (Figure 2(a)) after glucose injection, and blood insulin levels in TRIF^−/−^ mice were significantly higher than those in TRIF^+/+^ mice at 0 min (*P* < 0.05), 30 min (*P* < 0.001), 60 min (*P* < 0.05), and 90 min (*P* < 0.05) (Figure 2(b)). There is no difference in the ITT between the two groups (Figures 2(c) and 2(d)). In line with our observations, Hutton et al. [23] previously reported reduced glucose tolerance by GTT and normal ITT in TRIF^−/−^ mice. Based on pancreatic *β* cell morphology and insulin secretion from cultured *β* cells after stimulation with glucose in vitro, they concluded that TRIF is essential for maintaining normal *β* cell function and blood glucose homeostasis. They, however, did not investigate the effects of TRIF deficiency on food intake, bodyweight, and white adipose tissue in mice, whose changes can alter glucose metabolism.

**Figure 3:**
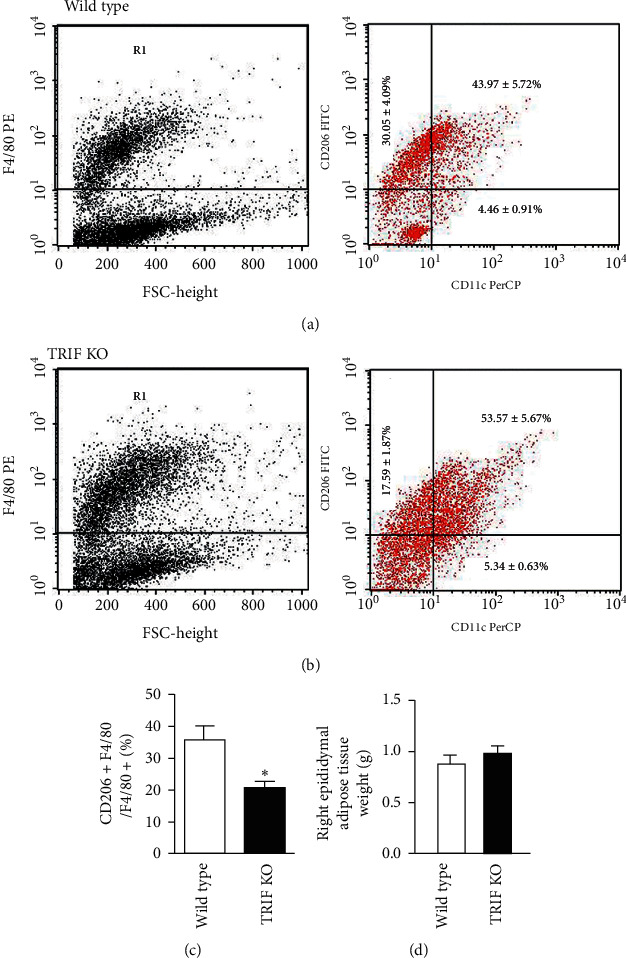
Effects of TRIF deficiency on macrophages in adipose tissue. (a) Flow cytometer analyses showing expression levels of CD206^+^ F4/80^+^ M2 and CD11c^+^ F4/80^+^ M1 macrophages in epididymal adipose tissue in wild-type mice (b) and TRIF KO mice. (c) Bar graphs present percentages of CD206^+^ F4/80^+^ M2 macrophages in F4/80^+^ macrophages. (d) The weights of right epididymal adipose tissue ( ^*∗*^*P* < 0.05) (*n* = 4/group).

White adipose tissue is the largest endocrine organ in most mammals, and epididymal adipose tissue, a part of white adipose tissue, plays essential roles in energy storage, endocrine communication, and insulin sensitivity/resistance [38]. Immune cells, including innate and adaptive immune cells in adipose tissues, play important roles in glucose metabolism. Inflammation in adipose tissues in obese subjects is characterized by a shift of immune cells from anti-inflammatory to proinflammatory state [39]. Notably, the major populations of adipose tissue macrophages in lean adipose tissue are M2 macrophages, which express high levels of arginase-1, the mannose receptor (CD206), and CD301 and secrete anti-inflammatory cytokines including the IL-10 and IL-1 receptor antagonist (IL-1Ra) [40]. We determined the percentage of CD206^+^ F4/80^+^ M2 macrophages in F4/80^+^ macrophage in epididymal tissues by FACS (Figures 3(a) and 3(b)). The percentages of CD206^+^ F4/80^+^ M2 macrophages (17.59 ± 1.87%) in F4/80^+^ cells in TRIF^−/−^ mice were lower than those (30.5 ± 4.09%) in TRIF^+/+^ mice (Figure 3(c), *P* < 0.05). However, several genetic mouse models have indicated that deletion of components of the TLR signaling pathway is associated with protection against white adipose tissue inflammation and/or rescue of metabolically perturbed phenotypes [41]. TRIF deficiency protects mice from lard-induced inflammation in adipose tissue and decreased insulin sensitivity [28] and from HFD-induced CD11c^+^ macrophage production in adipose tissue [26]. Unexpectedly, we found that TRIF deficiency decreases the percentage of M2 macrophages in epididymal adipose tissue. In addition, increased adipose mass increases the risk of developing insulin resistance and type 2 diabetes mellitus. These metabolic changes in obesity are accompanied by increased secretion of leptin from adipocytes as well as leptin resistance/insensitivity [42]. In this study, we found that the levels of plasma leptin are higher in TRIF^−/−^ mice (1175.2 ± 59.7 pg/ml) than those in TRIF^+/+^ mice (637.5 ± 100.8 pg/ml) (*P* < 0.01) (Figure 1(c)) although we did not find the difference in the weight of epididymal adipose tissues between the two groups (Figure 3(d)). It is possible that increased leptin counteracts food intakes and fat storage in adipose tissues in TRIF^−/−^ mice. Alternatively, TRIF^−/−^ mice are developing leptin resistance. These observations suggest that TRIF may play dual roles in regulating immune and metabolic homeostasis depending on the degree of metabolic stress under different diets such as a diet rich in saturated lipids.

**Figure 4:**
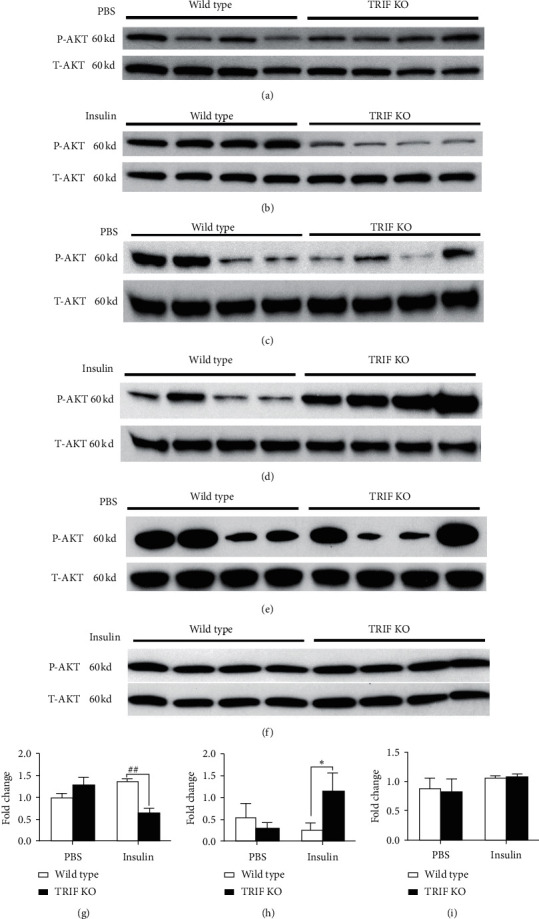
Effects of TRIF deficiency on activation of adipose tissue and liver AKT by insulin administration in mice. At 48 weeks of age, mice were fasted for 16 h and then intraperitoneally administered with PBS (a, c, e) or insulin (5 U/kg bodyweight) (b, d, f). Epididymal adipose tissue, muscle, and liver were collected 10 min after the administration. Levels of phospho-AKT (S473, P-Akt) and total-AKT (T-AKT) in adipose tissue (a, b), muscle (c, d), and liver (e, f) were determined by western blotting. (g–i) Bar graphs represent the results of densitometric analysis of western blots shown in (a) and (b), in (c) and (d), and in (e) and (f), respectively ( ^*∗*^*P* < 0.05,  ^##^*P* < 0.001) (*n* = 4/group).

Insulin resistance is associated with obesity and central components of type 2 diabetes and leads to altered glucose and lipid metabolism in adipose tissue, liver, and skeleton muscles. Insulin is indispensable for the normal development and function of adipose tissue [43]. The serine/threonine protein kinase (AKT) pathways participate in insulin signal transduction and insulin resistance [44, 45]. TLR4 is expressed in adipocytes as well as macrophages in adipose tissues [46, 47]. TLR4^−/−^ mice were shown to be more insulin sensitive in a lipid infusion model and more resistant to HFD-induced as well as lipopolysaccharide-induced insulin resistance [36, 48]. However, it is unclear that TRIF is involved in alterations in insulin sensitivity/resistance in TLR4^−/−^ mice. Here, we investigated activation of protein kinase B (PKB or AKT) in epididymal adipose tissue in response to insulin administration in TRIF^−/−^ mice. Phosphorylated AKT (pAKT) levels were significantly decreased in the adipose tissue of TRIF^−/−^ mice ten minutes after an intraperitoneal injection of insulin compared with those in TRIF^+/+^ mice (Figures 4(b) and 4(g), *P* < 0.001). No differences in pAKT levels were found between the two groups after PBS injection (Figures 4(a) and 4(g)). Because muscle and liver are also very important tissues in glycolipid metabolism, we investigated the activation of AKT in these two organs. In contrast with adipose tissue, we observed significant increases in pAKT in the muscle tissues of TRIF^−/−^ mice compared with that in TRIF^+/+^ mice after insulin injection (Figures 4(d) and 4(h), *P* < 0.001) but no difference between the two groups after PBS injection (Figures 4(c) and 4(h)), indicating that pAKT in muscle may not involve in glucose metabolism in response to insulin in TRIF^−/−^ mice. Indeed, skeletal muscle-specific AKT knockout mice show normal skeletal muscle insulin sensitivity and glucose tolerance [49]. The role of TRIF in skeletal muscle AKT signaling remains to be investigated in the future experiments. Such alterations in pAKT levels were not observed in liver of TRIF^−/−^ mice after PBS and insulin injection (Figures 4(e), 4(f) and 4(i)). Our findings indicate that insulin/AKT signaling in adipose tissue is decreased in TRIF^−/−^ mice but not in liver and muscle. These results suggest that impaired glucose tolerance in TRIF^−/−^ mice is due to decreased insulin/AKT signaling by proinflammatory changes in epididymal adipose tissues.

## 4. Conclusions

In conclusion, we found that TRIF deficiency in normal chow diet-fed mice increases food intake, plasma leptin levels, and bodyweight. TRIF^−/−^ mice show decreases in glucose tolerance and in M2 macrophages in epididymal adipose tissue. Insulin/AKT signaling in epididymal adipose tissue is impaired in TRIF^−/−^ mice, which can be explained by decreases in M2 macrophages. Jia et al. reported that hepatocyte-specific, but not myeloid cell-specific, TLR4-deficient mice were protected from HFD-induced white adipose tissue inflammation and insulin resistance in spite of obesity and suggested a hepatocyte TLR4-targeted approach as a useful therapeutic strategy of type 2 diabetes [33]. Here, we found that the TRIF-dependent TLR signaling contributes to maintaining insulin/AKT signaling and M2 macrophages in epididymal adipose tissue under a normal chow diet. Our study here provides new evidence that the TLR4-targeted treatment for type 2 diabetes requires caution.

## Data Availability

All data used to support the findings of this study are available from the corresponding authors upon request.
